# Resting Theta/Beta Ratios Mediate the Relationship Between Motor Competence and Inhibition in Children With Attention Deficit/Hyperactivity Disorder

**DOI:** 10.3389/fpsyg.2021.649154

**Published:** 2021-06-03

**Authors:** Chi-Fang Lin, Chung-Ju Huang, Yu-Jung Tsai, Ting-Yu Chueh, Chiao-Ling Hung, Yu-Kai Chang, Tsung-Min Hung

**Affiliations:** ^1^Department of Physical Education and Sport Science, National Taiwan Normal University, Taipei, Taiwan; ^2^Graduate Institute of Sport Pedagogy, University of Taipei, Taipei, Taiwan; ^3^Department of Physical Education and Sport Science, National Taiwan Normal University, Taipei, Taiwan; ^4^Department of Athletics and Master Program of Sport Facility Management and Health Promotion, National Taiwan University, Taipei, Taiwan; ^5^Department of Physical Education and Sport Science, Institute for Research Excellence in Learning Science, National Taiwan Normal University, Taipei, Taiwan

**Keywords:** ADHD, TBR, motor ability, interference, mediator

## Abstract

Despite that previous studies have supported relationships between motor ability and inhibitory function, and between resting brain theta/beta power ratios (TBR) and inhibition in children with attention deficit/hyperactivity disorder (ADHD), little research has examined the mechanism within these relationships. The purpose of this study was to investigate whether TBR would mediate the relationship between motor ability and inhibitory function. A total of 71 children with ADHD were recorded resting electroencephalographic (EEG) data during eyes-open. Motor abilities were evaluated by Movement Assessment Battery for Children-2 (MABC-2) and inhibitory ability were assessed by a modified Eriksen’s flanker task. The results of mediation analyses revealed that TBR could completely mediate the relationship between motor competence and response speed (indirect effect = −0.0004, 95% CI [−0.0010, −0.0001]) and accuracy (indirect effect = 0.0003, 95% CI [0.0000, 0.0010]) in the incongruent condition of the flanker task. This study suggests that TBR may be one of the mechanisms between motor ability and inhibition function in children with ADHD.

## Introduction

Attention-deficit/hyperactivity disorder (ADHD) is one of the most pervasive childhood developmental disorders and approximately 27% of children with ADHD suffer from deficits in inhibition function (Kofler et al., 2019). Inhibition refers to the ability to inhibit responses, which is associated with executive neuropsychological functions that appear to depend on it for their effective execution such as working memory, regulation of motivation, internalization of speech, and reconstitution (Barkley, 1997), as well as fine and gross motor abilities (Band and van Boxtel, 1999). Inhibitory dysfunctions typically result in long-term consequences as problems in mood regulation, family life, peer relationships, academic achievement and future prospects among children with ADHD (Youn et al., 2019). Although medication and behavioral treatments for ADHD symptoms are recommended, some potential side effects and costs often reduce their overall effectiveness (Rajeh et al., 2017) and thus making it important to explore lifelong adjunctive or alternative treatment options that may benefit children with ADHD. Based on this concern, factors associated with inhibition functions in children with ADHD and their mechanisms deserve further investigation.

For children with ADHD, problems of fine and gross motor skills, coordination skills, and motor control have been found to relate to inattentive or combined symptoms (Fenollar-Cortés et al., 2017). Therefore, deficits in motor competence are often accompanied by cognitive impairments in children with ADHD. Motor competence refers to the degree of proficiency in performing a wide variety of motor skills including both gross (e.g., jumping) and fine (e.g., manual dexterity or precision) motor skills as well as the underlying mechanisms including coordination, control, and quality of movement. There is extensive research examining potential relations between motor competence and inhibition functions in different populations. Recent research within typically developing children has reported a significant relationship between motor competence and response inhibition (Albuquerque et al., 2021), and children with typical development exhibit better inhibitory performance than children with motor impairments (Yu et al., 2020). Also, motor competence such as coordination associated with throwing and striking and hand dexterity could predict inhibitory performance at a Go/No-Go task in children with ADHD (Hung et al., 2013). Several intervention studies have identified beneficial effects of motor skill training programs on inhibition and attention in children with ADHD. For example, Verret et al. (2012) found that a 10-week ball games program (basketball and soccer) not only improved fitness and gross motor skills but also information processing and sustained auditory attention. Similarly, Pan et al. (2016) reported a 12-week table tennis exercise program improved motor skills (e.g., locomotor and object-control skills) and performance on tasks involving planning and inhibition. Furthermore, the relationship between motor competence and inhibition function appears to be causal since it has been shown that an 8-week coordination exercise program led to improved inhibition in kindergarten children (Chang et al., 2013). Previous research has indicated that common mechanisms associated with the cognitive deficits and motor difficulties of children with ADHD may involve several brain structures (Halperin and Healey, 2011) and neurotransmitter systems (Prince, 2008). Therefore, high levels of motor competence might contribute to enhanced inhibition via the restoration of normal functioning involving these brain areas and neurotransmitter systems.

Motor cortical regions of the frontal cortex are considered to be highly associated with the acquisition and/or consolidation of skilled motor behaviors, as well as rich dopaminergic pathways (Luft and Buitrago, 2005). Furthermore, by using a technique of transcranial magnetic stimulation, short interval cortical inhibition (SICI) evoked in motor cortex is significantly reduced in children with ADHD compared to typically developed children (Gilbert et al., 2011). Reduced SICI in motor cortex is viewed to correlate with the presence and severity of hyperactive impulsive behaviors in children with ADHD as well as with commonly observed delays in motor control. Therefore, the neural substrate of these motor delays and inhibition dysfunction may include mechanisms within or adjacent to the motor cortex. Given that children with ADHD are commonly featured by motor difficulties and inhibitory control deficits, the association between low motor competence and weak inhibition might be mediated by some stable and predisposed variables. A resting EEG indicator, theta/beta ratios (TBR), might be one of the mediators. Inhibition dysfunction in children with ADHD is identified to be resulted from cortical under-arousal (Clarke et al., 2001) as indicated by increased ratios of EEG slow waves to fast waves, such as TBR, during the resting state. Previous research has demonstrated a negative relationship between TBR and inhibition performance (Putman et al., 2010) and suggested that TBR may serve as a stable biomarker for inhibition in children with ADHD (Zhang et al., 2017). Moreover, in a randomized controlled trial, neurofeedback training aimed at reducing TBR resulted in reduced symptoms in children with ADHD (Janssen et al., 2016). Regarding the association between motor competence and TBR, previous research has found that children with ADHD show worse motor competence and higher TBR than typical development children (Huang et al., 2018). Based on maturational lag and hypo-arousal model, elevated TBR in children with ADHD is partly associated with delayed development of the brain’s ascending reticular activation system that leads a disturbance in thalamus-cortical transactions (Saad et al., 2018). By using a sample of normal adult mice, the acquisition of motor skills has been found to induce synaptic modifications in the primary motor cortex in which the stabilization of motor thalamus can be enhanced (Hasegawa et al., 2020). Although little evidence has directly shown the causal relationship between motor competence and TBR, high levels of motor competence may contribute to develop more efficient thalamocortical pathways which may be reflected by reduced TBR. Therefore, it is postulated that a negative association between motor competence and TBR might be expected in children with ADHD.

While a positive relationship has been demonstrated between motor competence and inhibition in children with ADHD, there is limited research as to the underlying mechanism (Chang et al., 2013; Hung et al., 2013). Given that motor competence (Halsband and Lange, 2006), TBR (Chabot and Serfontein, 1996), and inhibition (Casey et al., 1997) all involve the frontal cortex and it is known that reduce in TBR may reflect improved inhibition, it is possible that the influence of motor competence on inhibition is mediated through the biological/physiological pathways reflected by TBR. Therefore, the main purpose of this study was to examine whether TBR could mediate the relationship between motor competence and inhibition in children with ADHD.

## Method

### Participants

A total of 71 children with ADHD (mean age = 9.90 ± 1.54 years) from local elementary schools in Taipei were included in this study (Table 1). They were referred by special education teachers and parents to participate in this study. Sample size was determined to ensure sufficient power by using power analysis software (G^∗^Power 3.1). We set the following input parameters for using a linear multiple regression with alpha = 0.05, power = 0.80, effect size *f*^2^ = 0.15 (medium), and 2 predictors. The resulting sample size specification was 68. Therefore, the current sample was shown to have a greater than 80% probability of detecting a medium size effect. Recruitment criteria were as follows: (1) aged between 8 and 12 years; (2) diagnosed by a pediatric psychiatrist as having ADHD based on the criteria of the fifth edition of the *Diagnostic and Statistical Manual for Mental Disorders* (DSM-5; American Psychiatric Association, 2013); (3) no co-morbid conditions, such as conduct/oppositional defiant disorder, autism spectrum disorders, or serious affective disorders; and (4) no history of brain injury or neurological conditions, such as epileptic seizures, serious head injuries, or periods of unconsciousness. In addition, the legal guardians of our participants confirmed the presence of the ADHD symptoms using the Child Behavior Checklist (CBCL), a component in the Achenbach System of Empirically Based Assessment (Achenbach and Rescorla, 2001). For the purpose of this study, only the items relevant to the assessment of ADHD were utilized. The average ADHD scale score for the participants in the current study was 67.18 ± 6.8. While the clinical cutoff score for a diagnosis of ADHD is above 70 (Achenbach and Rescorla, 2001), score ranging from 65 to 69 are considered clinically borderline. All participants were instructed to stop medications for at least 24 hours prior to the start of this study. Their parents completed a written assessment and gave informed consent. This study was approved by the Institutional Review Board of National Taiwan Normal University (201010HS001).

**TABLE 1:** Demographic and physical characteristics of the participants.

Characteristics	Mean ± SD
Gender (boy: girl)	48:23
BMI (kg/m^2^)	19.50 ± 1.5
ADHD type	
ADHD-I	30
ADHD-HI	5
ADHD-C	36

*BMI, body mass index; ADHD-I, predominantly inattentive subtype; ADHD-HI, predominantly hyperactive-impulsive subtype; ADHD-C, combined hyperactive-impulsive and inattentive subtype.*

### Measure

#### Motor Competence

This study used the Movement Assessment Battery for Children-2 (MABC-2) to assess participants’ motor abilities, a test designed to identify and describe impairments in motor performance of children and adolescents 3 through 16 years of age (Henderson et al., 2007). The MABC-2 includes 3 subtests: manual dexterity (placing pegs, threading and drawing), aiming and catching (two handed catching and beanbag throwing), and balance (on-board balancing, walking heel to toe, and hopping). The three subsets of the MABC-2 were converted into standard scores, then transform to the percentile scores on these subsets. Although some items of the MABC might need to be adjusted in Asia-pacific countries (Chow et al., 2006), the reliability and validity of the MABC-2 are still fair based on a large sample of Chinese preschool children (Hua et al., 2013).

#### Inhibition

The Eriksen’s flanker task was used to examine interference control and inhibition. The task included two types of trials. In ‘congruent’ trials stimuli consisted of a horizontal array of five arrows all facing in the same direction (i.e., <<<<< or >>>>>). In ‘incongruent’ trials, the central target arrow was flanked by two arrows on each side facing in the opposite direction (i.e., <<><< or >><>>). By replicating the protocol used in a previous study (Tsai et al., 2017), the flanking stimuli were presented as 3 × 3-cm figures, at a 2.87° visual angle, in the center of a computer screen, 60 cm from the participant, on a black background. Participants completed 5 blocks of 32 trials presented with equiprobable congruency and directionality. Left and right target arrows were presented with equal probability and were randomized within each block. Each trial began with a central fixation cross (+) for 1000 ms, followed by the presentation of the target stimulus in the center of the computer screen for 200 ms. A blank screen was then shown for 1500 ms. The next trial began after a response was made, or at the end of the response window, i.e., 1700 ms after the target stimulus presentation. Participants were instructed to respond to the central target arrow by pressing the keyboard letters ‘V’ (with their left index finger) or ‘N’ (with their right index finger) as quickly and accurately as possible, to indicate whether the stimulus presented pointed to the left or right respectively. Participants were allowed a one-minute break between blocks. The total duration of the task was approximately 12 min.

The behavioral performance measures including reaction time (RT) and accuracy (correct responses) were calculated in the congruent and incongruent trials, respectively. RT was defined as the interval between the onset of a stimulus and a response. Only RTs recorded from correct responses occurring within the response window (between 150 ms and 1000 ms) were included for subsequent analysis.

### EEG Recording and Data Reduction

Electroencephalographic activity was recorded from 28 midline and lateral Ag/AgCl electrodes positioned according to the International 10–20 system using a NeuroScan Quik-Cap (Neuro Inc., Charlotte, NC, United States). Vertical electrooculogram (VEOG) was collected from electrodes placed above and below the left eye and the horizontal electrooculogram (HEOG) from the outer canthus of each eye. A ground electrode was attached to the middle of the forehead, and all electrodes were referenced to linked ears. Electrode impedance was kept below 10 kΩ. EEG and EOG were amplified with a bandwidth of DC-to-100 Hz. The sampling rate was 500 Hz. In addition, a 60-Hz notch filter was utilized during the data acquisition. Prior to data processing, EEG data were filtered using a band-pass cutoff of 1-30 Hz (12 dB/octave) and EOG corrected by an algorithm. The continuous EEG data were segmented into 2-s epochs. After a baseline correction and visual inspection for artifact, the cleaned EEG data were Fast Fourier transformed and subsequently ln-transformed. Power estimates were calculated using fixed frequency (4-7.5 Hz for theta and 13.5-25 Hz for beta) bands at the Cz site, which has been found to have the greatest ability to distinguish between ADHD and healthy controls (Monastra et al., 1999). Furthermore, the ratio coefficient of theta/beta was computed.

### Procedure

Each participant came into the laboratory on a single occasion. The day before participants were reminded to avoid caffeine and food products containing alcohol. On arrival in the laboratory, participants were familiarized with the testing procedure as well as the laboratory environment. Written informed consent forms, as well as health and demographic questionnaires were provided to the accompanying adults. Participants were then asked to complete a test of motor competence, after which they were fitted with an electrode cap, and impedances and the quality of the EEG signal were checked. Then, sitting calmly in a comfortable chair with eyes open in a sound-attenuated testing room, EEG data was collected twice for one minute each. After these two EEG data collections, the participants then engaged in the flanker task.

### Statistical Analysis

Descriptive statistics were computed, and Pearson correlation coefficients were estimated among motor competence (the MABC-2 score), the resting TBR, and inhibition indicators (RT and accuracy rate at the flank task). A bootstrapping method was performed using SPSS PROCESS Macro (Hayes, 2013) to examine if the resting TBR mediated the relationship between motor competence and behavioral performance of the flanker task in children with ADHD. The mediation analyses were conducted with 1,000 bootstrapping resamples to obtain bias-corrected 95% confidence intervals. All statistical analyses were performed using SPSS 21.0 with a significance level of 0.05.

## Results

### Preliminary Analysis

As shown in Table 2, motor competence (the MABC-2 score) was negatively correlated with the TBR, RT in the congruent trials (C-RT), and RT in the incongruent trials (IC-RT), as well as positively correlated with accuracy in the congruent trials (C-ACC) and accuracy in the incongruent trials (IC-ACC). Regarding the subsets of the MABC-2, the percentile score of manual dexterity was negatively correlated with C-RT and IC-RT, as well as positively correlated with C-ACC and IC-ACC. The percentile score of aiming and catching was negatively correlated with the TBR and positively correlated with C-ACC. The TBR was negatively correlated with IC-ACC and positively correlated with C-RT and IC-RT. All significant levels reached at *p* < 0.05.

**TABLE 2:** Correlations among Motor Competence, TBR, and Inhibitory Performance.

	1	2	3	4	5	6	7	8	9
1. MABC-2 (%)	-								
2. Manual dexterity (%)	0.71**	-							
3. Aiming & catching (%)	0.78**	0.36**	-						
4. Balance (%)	0.59**	0.12	0.31**	-					
5. TBR (ratio)	-0.25*	-0.11	-0.27*	-0.18	-				
6. C-RT (sec.)	-0.32**	-0.28*	-0.19	-0.14	0.35**	-			
7. C-ACC (%)	0.38**	0.38**	0.27*	0.02	-0.14	-0.15	-		
8. IC-RT (sec.)	-0.29*	-0.29*	-0.14	-0.13	0.32**	0.95**	-0.11	-	
9. IC-ACC (%)	0.29*	0.32**	0.23	0.06	-0.30**	-0.07	0.69**	-0.12	-

M	56.93	52.99	55.09	56.82	2.67	0.57	0.88	0.66	0.76
*SD*	26.20	26.41	29.57	26.75	0.40	0.13	0.08	0.16	0.15

*p < 0.05 and **p < 0.01. MABC-2, average percentile of the three subsets of the Movement Assessment Battery for Children-2; TBR, theta/beta ratio; C-RT, RT in the congruent condition; C-ACC, accuracy rate in the congruent condition; IC-RT, RT in the incongruent condition; IC-ACC, accuracy rate in the incongruent condition.

### Mediation Analyses

The purpose of this study aimed to examine whether TBR could mediate the relationship between motor competence and inhibition in children with ADHD. A bootstrapping method was performed using SPSS Process Macro to conduct the mediation analyses. As shown in Table 3, the motor competence (independent variable) could significantly predict C-RT of the flanker task (dependent variable), and the resting TBR (mediator). Next, the resting TBR was a significant predictor of C-RT of the flanker task. While controlling for resting TBR, the result showed that the motor competence was still a significant predictor of C-RT of the flanker task. The results of the indirect effect based on 1000 bootstrap samples revealed that a significant indirect negative relationship between motor competence and C-RT of the flanker task was partially mediated by the resting TBR.

**TABLE 3:** Mediation Analysis for RT in the Congruent Trials of the Flanker Task (C-RT).

Variable/Effect	*b*	*SE*	*t*	*p*	*95% CI*	
MC→TBR	−0.0038	0.0018	−2.10	0.0391	−0.0074	−0.0002
TBR →C-RT	0.0949	0.0372	2.55	0.0130	0.0206	0.1691
MC→C-RT	−0.0012	0.0006	−2.16	0.0340	−0.0024	−0.0001

**Effect**						
Direct	−0.0012	0.0006	−2.16	0.0340	−0.0024	−0.0001
Indirect	−0.0004	0.0002			−0.0009	−0.0000
Total	−0.0016	0.0006	−2.77	0.0072	−0.0028	−0.0004

*MC, motor competence; TBR, theta/beta ratio; CI, confidence interval.*

As shown in Table 4, the motor competence was significant predictors of C-ACC of the flanker task and the resting TBR. However, the resting TBR was not a significant predictor of C-ACC of the flanker task. The resting TBR played no role in the relationship between motor competence and C-ACC of the flanker task.

**TABLE 4:** Mediation analysis for accuracy rate in the congruent trials of the flanker task (C-ACC).

Variable/Effect	*b*	*SE*	*t*	*p*	*95% CI*	
MC→TBR	−0.0038	0.0018	−2.10	0.0391	−0.0074	−0.0002
TBR →C-ACC	−0.0111	0.0254	−0.44	0.6631	−0.0617	0.0395
MC→C-ACC	0.0012	0.0004	3.17	0.0023	0.0005	0.0020

**Effect**						
Direct	0.0012	0.0004	3.17	0.0023	0.0005	0.0020
Indirect	0.0000	0.0001			−0.0001	0.0003
Total	0.0013	0.0004	3.40	0.0011	0.0005	0.0020

*MC, motor competence; TBR, theta/beta ratio; CI, confidence interval.*

As shown in Tables 5, 6, the motor competence could significantly predict IC-RT and IC-ACC of the flanker task and the resting TBR. Next, the resting TBR was a significant predictor of IC-RT and IC-ACC of the flanker task, respectively. While controlling for resting TBR, the result showed that the motor competence could not significantly predict IC-RT and IC-ACC of the flanker task. The results of the indirect effect indicated that the resting TBR could completely mediate a significant indirect negative relationship between motor competence and IC-RT, as well as a significant indirect positive relationship between motor competence and IC-ACC of the flanker task.

**TABLE 5:** Mediation Analysis for RT in the Incongruent Trials of the Flanker Task (IC-RT).

Variable/Effect	*b*	*SE*	*t*	*p*	*95% CI*	
MC→TBR	−0.0038	0.0018	−2.10	0.0391	−0.0074	−0.0002
TBR →IC-RT	0.1059	0.0465	2.28	0.0259	0.0131	0.1987
MC→IC-RT	−0.0014	0.0007	−1.92	0.0593	−0.0028	0.0001

**Effect**						
Direct	−0.0014	0.0007	−1.92	0.0593	−0.0028	0.0001
Indirect	−0.0004	0.0002			−0.0010	−0.0001
Total	−0.0018	0.0007	−2.48	0.0155	−0.0032	−0.0003

*MC, motor competence; TBR, theta/beta ratio; CI, confidence interval.*

**TABLE 6:** Mediation Analysis for Accuracy Rate in the Incongruent Trials of the Flanker Task (IC-ACC).

Variable/Effect	*b*	*SE*	*t*	*p*	***95% CI***	
MC→TBR	−0.0038	0.0018	−2.10	0.0391	−0.0074	−0.0002
TBR →IC-ACC	−0.0918	0.0429	−2.14	0.0359	−0.1774	−0.0062
MC→IC-ACC	0.0013	0.0007	1.96	0.0530	0.0000	0.0026

**Effect**						
Direct	0.0013	0.0007	1.96	0.0530	0.0000	0.0026
Indirect	0.0003	0.0002			0.0000	0.0010
Total	0.0017	0.0007	2.51	0.0145	0.0003	0.0030

*MC, motor competence; TBR, theta/beta ratio; CI, confidence interval.*

## Discussion

The purpose of the present study was to explore the relationships among motor competence, TBR, and inhibition function in children with ADHD. Results showed a positive association between motor competence and inhibition, whereas negative associations of TBR with motor competence and inhibition. More importantly, this study found that TBR could mediate the relationship between motor competence and inhibition. Specifically, TBR completely mediated the relationship between motor competence and response speed and accuracy in the incongruent condition of the flanker task, and partially mediated the relationship between motor competence and response speed in the congruent condition.

The results showed that motor competence was positively correlated with inhibition, which is consistent with previous findings indicating that higher motor competence is associated with better inhibition function and cognitive flexibility in children with ADHD (Hung et al., 2013; Chang et al., 2014; Pan et al., 2016). Even a single session of combined motor coordination and aerobic exercise could improve inhibition and the allocation of attentional resources in children with ADHD (Ludyga et al., 2017). Evidence from healthy individuals also indicates that motor competence is positively associated with perceived competence and multiple aspects of health including physical activity, cardiorespiratory fitness, muscular strength, muscular endurance, and a healthy weight status (Utesch et al., 2019). For example, Wu et al. (2017) showed that infants’ gross motor ability assessed at 1 or 2 years old predicted cognitive inhibitory control and working memory performance 2 years later. Early developmental delay of motor competence has been found to negatively impact or coexist with additional developmental domains (e.g., cognitive, social) and/or lead to long-term health-related consequences (Brian et al., 2019). Therefore, the longitudinal impact of motor competence in early childhood should be emphasized as motor competence may influence cognitive, social, and health domains from different pathways. This is possibly due to shared neural substrates as previous research has shown that basal ganglia, the cerebellum, and the prefrontal cortex are co-activated in top-down control of behavior in both complex cognitive and motor tasks (Leisman et al., 2016).

The present study found that TBR was negatively correlated with inhibition. This finding is compatible with studies employing a Go/No-Go task (Putman et al., 2010), a flanker task (Zhang et al., 2017), and the Attentional Control Scale (Putman et al., 2014) to assess inhibition in children with ADHD or healthy individuals. Elevated theta, decreased beta, and increased TBR during the resting stage in children with ADHD may reflect a dysfunction in the frontal cortical regulation of subcortical processes (Schutter and van Honk, 2006). Compared to controls, ADHD was associated with disrupted connectivity between within-default mode network and cognitive control and affective/motivational and salience networks (Sutcubasi et al., 2020). This dysfunction may lead to inattention symptoms and inhibitory control deficits presented by a series of cognitive indicators such as increased omission errors, slower RTs, and elevated RT variability (Gambin and Święcicka, 2009). As such, a negative association between TBR and inhibition is to be expected. The present result also revealed a negative correlation between motor competence and TBR. Although no existent research identifies the influence of motor competence on TBR, Huang et al. (2018) reported that children with ADHD exhibited lower motor competence and higher TBR than typical development children. As high levels of motor competence are linked to both high working memory maintenance and effective task preparation in adolescents (Ludyga et al., 2018), the potential role of motor competence in promoting positive or negative trajectories of brain function should be noticed. Our result can be explained by the fact that high levels of motor competence is beneficial to the function of motor cortical regions such as frontal cortex and cerebellum (Halsband and Lange, 2006), which are under-aroused during the resting state in children with ADHD (Clarke et al., 2001). That is, a higher level of motor competence implies better development of these brain regions reflected by more normalized TBR.

In support of our hypothesis, inhibition, measured by response accuracy and RT at the flanker task, was predicted by motor competence, and this relationship was mediated by TBR. Previous studies indicated that ADHD-related deficits are associated primarily with the frontal-striata and frontal-parietal network (Durston et al., 2003), which are also activated during tasks requiring motor skill execution and inhibition. Therefore, high motor competence may be accompanied by improved inhibition in children with ADHD; meanwhile, biological/physiological pathways in the frontal cortex are frequently activated during the process of establishing motor competence. This positive development of neurophysiological pathways in the frontal cortex can be reflected in reduced resting TBR. Specifically, TBR was entirely responsible for the relationship between motor competence and inhibition in the incongruent condition of the flanker task, whereas TBR accounted for only part of this relationship in the congruent condition. The differences could be due to a higher demand of inhibition function in the incongruent condition. Previous studies have shown that children with ADHD make relatively more errors in the flanker task than normally developing children, especially in the incongruent condition (Crone et al., 2003). While conducting tasks exerting more complex demands on the inhibitory ability, individuals are required to make increased activation in the inferior frontal cortex, dorsolateral prefrontal cortex, and the pre-supplementary motor area to achieve the tasks (Criaud and Boulinguez, 2013). On the other hand, task performance in the congruent condition may be affected by brain regions other than the frontal cortex due to there being less demand on executive function. For individuals who have higher motor competence, they may exhibit fewer inattentive symptoms reflected by reduced resting TBR. Therefore, in more complex tasks, they are more likely to mobilize relevant cognitive resources to meet the high levels of cognitive demand. The mediating role of TBR in the relationship between motor competence and inhibition in children with ADHD is becoming increasingly apparent under this situation. These ideas are speculative and future work is required before any definitive conclusions can be drawn.

Caution should be exercised when interpreting the results of this study. First, although the results found possible relationships among motor competence, resting TBR, and inhibition, experimental studies are needed to provide stronger evidence for the causal nature of this relationship. Second, the number of correct trials in the incongruent condition would be too small to calculate RT appropriately. Although the current study followed the research protocol used in Tsai et al. (2017), a smaller number of trials and higher error rate in the incongruent condition may result in inaccurate estimate of RT. Third, given that increased TBR and weaker inhibition function in ADHD populations compared with healthy control groups have been identified, this study did not utilize a sample of normally developing children as a control group. The inclusion of such a group would enable a determination of the extent to which the mediating effect of TBR in the relationship between motor competence and inhibition is specific to children with ADHD, or if it reflects a general relationship between motor competence and inhibition regardless of whether ADHD is present. In summary, the current study found that inhibition is predicted by motor competence, and this relationship is mediated by TBR in children with ADHD. This finding has significant implication in the interpretation of the relationship between motor competence and inhibition function in children with ADHD. Also, given that inhibition is thought to play an important role in mood regulation, social relationships, academic learning and future development in children with ADHD, participation in physical exercise aiming at improving both motor abilities and physical fitness may have greater implications for their development. For future researchers, our findings suggest that other mediators could exist and need to be further explored. It is also recommended that future studies look at the relationship of different facets of motor competence to subcomponents of executive functions in order to describe this relationship in more detail.

## Data Availability Statement

The raw data supporting the conclusions of this article will be made available by the authors, without undue reservation.

## Ethics Statement

The studies involving human participants were reviewed and approved by The Institutional Review Board of National Taiwan Normal University. Written informed consent to participate in this study was provided by the participants’ legal guardian/next of kin.

## Author Contributions

C-FL and C-JH were responsible for the research idea, implementing the study, and manuscript writing up. C-LH was responsible for the statistical support and discussion commentary. Y-JT and T-YC were responsible for assisting on the data collection and analysis. Y-KC was responsible for consulting the methodology and interpretation of the findings. T-MH was responsible for the discussion of the research idea, supervision of the data collection, and comment on the manuscript writing up. All authors contributed to the article and approved the submitted version.

## Conflict of Interest

The authors declare that the research was conducted in the absence of any commercial or financial relationships that could be construed as a potential conflict of interest.
